# Student Writing Weekends: A Model for Encouraging Undergraduate Student Publication

**DOI:** 10.3389/fpsyg.2019.01183

**Published:** 2019-05-24

**Authors:** Rhoda Scherman

**Affiliations:** Department of Psychology, Faculty of Health and Environmental Sciences, Auckland University of Technology, Auckland, New Zealand

**Keywords:** writing retreat, tertiary student, publication, research dissemination, undergraduate, mentoring, supervision, contributing to the greater good

“*Research should be alive and shared, it should lead to something. Publishing research outputs in scholarly journals will continue to have value if these outputs add to our knowledge and thereby move the field ahead. Rather than relying on published works as the final evidence of impact, professors should be challenged to develop agency for passing on what they know to the next generation of scholars”* (Meyer, 2012, p. 216).

## Rationale for Undergraduate Publication

Meyer (2012) succinctly articulates the importance of publishing research—for advancing knowledge, which benefits the academic and those in her field. Moreover, Meyer argues that as academics, through our research and dissemination, we also have a responsibility to contribute to the social good. I fervently support this idea—as do others (e.g., Parija and Kate, 2017), and I would add that as researchers and supervisors, we have the added responsibility to help our students achieve the same goal. Yet, for most students, graduation and getting a job are the goals of tertiary study. Many undergraduate students do not think about publishing (Pittam et al., 2009), and may lack opportunities for publication (Weiner and Watkinson, 2014), as well as the skills, confidence and motivation to publish (Aitchison and Lee, 2006; Kamler, 2008; Maher et al., 2008).

Research tells us that *writing retreats* are an efficient and widely utilized model of enhancing academic publication (Grant, 2006; Singh, 2012; Dowling et al., 2013). They are based on a model of bringing together people who share the same goals of writing, in a live-in space for a specified number of days. Writing retreats usually involve a mix of individual and group activities, with large chunks of time dedicated to writing, in either communal or private spaces. Depending on the setting, meals may be provided, further decreasing distractions of daily life. In this way, writing retreats have been found to address many of the known barriers to publication (e.g., dedicated and protected time to write; removal of work and family distractions). They also improve collegiality, provide pastoral support, and are cost-effective. Using writing retreats for post-graduate students builds confidence (Aitchison, 2009; Jackson, 2009), is motivating and furthers employment (Petrova and Coughlin, 2012), and is said to be satisfying and productive (MacLeod et al., 2012).

Armed with a passionate belief that students share in the social responsibility to disseminate their research findings; that with instruction and support, students have the ability to publish; and a robust body of knowledge on the benefits of writing retreats to improve academic publication, I developed a model for encouraging undergraduate dissemination through supervisor-led *Student Writing Weekends (SWW)*. Below I describe the SWW, interspersed with reflexive comments on what worked, where changes were needed going forward, and some tips for anyone wishing to develop their own student writing weekends.

## Description of the Student Writing Weekends

For me, creating a space where students could work individually and collaboratively was essential to the success of the SWW. So, too, was working in an environment where distractions to writing were minimized. Lastly, I felt that some consecutive days of writing and instruction were needed in order to create momentum.

## Location

Staying overnight in my home achieved all three objectives. I acknowledge, however, that the location, layout and number of rooms were major factors in my ability to host the SWW. I further accept that some readers will be uncomfortable having students in their homes. As an alternative, Jackson (2009) holds larger writing retreats for post-graduate students at locations like hotels where students can share rooms, and where they will also have few distractions. If a campus has student housing, that could be another possibility. Additionally, if one's university is willing to share in the costs (and/or the students themselves), one can hire a venue. Ultimately, the location will not be as important as the features needed to mimic the “writing retreat” format. This would include overnight accommodation (for momentum and immersion); quiet spaces for writing; communal spaces for talking, collaborating or instructing; a kitchen for preparation of meals (if not provided by the venue); and in a geographical location away from external distractions. Finally, while I prefer students overnight so they maximize writing time and do not lose time traveling, it could be worth trialing consecutive day-only writing retreats. Modern university libraries, with their individual and shared work spaces, might offer the ideal location for such a format.

## Spaces Inside the House

Common sitting areas were labeled as *quiet-spaces* where students could focus and write. The kitchen and my home-office were both *talking-spaces*, with the later where students met with me to read over drafts and discuss projects. Sleeping arrangements were fairly arbitrary, often dictated by order of arrival. My home has two extra bedrooms. It also has two common sitting areas, each with furniture to sit on for writing, which also converted to sleeping surfaces at night.

## Structure and Format of Activities

For the first SWW, students were asked to arrive by 10 a.m. on Saturday morning, at which time we would have introductions, set guidelines for the weekend, and review some writing and publishing conventions. Unfortunately, several students arrived late, complicating these plans. So as not to delay the work of those already there, introductions had to be made *ad hoc*. In hindsight, this was problematic to workflow due to periodic interruptions as new people arrived. For SWW#2, students were asked to arrive on the Friday night before; this way, everyone could meet over dinner, settle into their spaces in the house, and awaken on Saturday morning prepared to start right into their work. For subsequent SWW, if students already knew one another, we returned to the Saturday morning start; if new students were joining, students were asked to arrive Friday night.

For me, the goal of the SWW was to build capacity, offer instruction, model the processes, and support the students through the shared experience of a writing retreat. As such, when it came to writing, I chose to let the students organize their writing times and activities as suited them—not “crack-a-whip.” Some readers will find this too relaxed, and upon reflexion, I would agree that more prescribed structure would be valuable.

The students were also urged to “make themselves at home,” so they would awaken and eat their morning and mid-day meals as suited them. The evening meal, on the other hand, was always prepared and eaten as a group, which brought us back together and offered some shared time to debrief about the day. During dinner, we would discuss writing projects, tackle any writing issues, and plan out writing for the next day. As needed, I would also give additional instruction on writing or publication. After dinner, anyone wishing to continue writing could do. Some did, but most chose to relax, talk, or play games—which served to strengthen the collegial atmosphere.

## Costs

The novelty and uncertainty of the first SWW incited me to cover food costs associated with the entire weekend. In subsequent SWW, students brought their own food for breakfast, lunch and snacks, and contributed something to the shared dinner. I would provide basics like tea, coffee, milk, bread, butter, etc., and the Friday night meal, when it occurs, is always provided by me. Others wishing to develop their own SWW might consider a small fee that covers the purchase of food for the group. However, the current system of having some shared and some personal food has worked well, and would likely be the best format for alternative retreat locations that lack dedicated food preparation services.

## Characteristics of the Students

Over the years, I have had from two to six students at any single SWW. They were all psychology students that I supervised in some research capacity. Most were former fourth-year Honors degree students who I supervised for their dissertations; others had taken an “independent study” class with me, during which time the students carried out small research projects or systematic literature reviews. Anyone who had previously worked with me in those contexts was welcomed with no further vetting criteria other than the desire to publish their work.

Most SWW attendees were only a year out from the previous research projects, meaning that virtually all were attempting to publish from earlier research, as they undertook further study. This fact created unique challenges that influenced the development—and outcomes—of the SWW. For instance, in light of their ongoing study, the students had only their semester breaks available for writing on previous projects. Unfortunately, many students needed those breaks to complete current assessments, preventing them from attending. For those who did attend, it meant taking a mental break from their current work, to reconnect to their former projects—a task of compartmentalizing that turned out to be too challenging for some of my students.

## Publications Achieved

While I believe that my students have a responsibility to disseminate, I do not believe it must only be in scholarly journals. That objective is neither achievable for every student, nor always desired; non-scholarly mediums are sometimes preferable, in order to reach one's stakeholders. Additionally, non-scholarly publications can be more achievable but equally rewarding for the students.

Since starting the retreats in 2014, my students and I have co-published a journal article (Jamieson and Scherman, 2014), book review (Scherman and Prakash, 2016), two magazine articles (Mousa and Scherman, 2014; Prakash and Scherman, 2016), and delivered two presentations (Scherman and Mousa, 2013; Scherman et al., 2013). Notwithstanding those successes, no manuscripts were completed in a single weekend, so the SWW served mainly to jump-start writing. For some, this was enough motivation to prioritize the manuscript among other coursework. For many, however, the time-constraints associated with ongoing coursework remained a real impediment to publishing.

## Going Forward

Upon reflection, there were two major barriers to more consistently publishing with my undergraduate students: (1) trying to reconnect to the former projects in the face of new coursework and assessments; and (2) the added challenge of having too little time in which to complete a manuscript for publication, if being done in the semester breaks. In trying to resolve these challenges, I have wondered if the writing that served to meet the academic requirements of the course or assessment, could be altered to more closely mimic the planned manuscript. Sadly, the learning outcomes associated with different coursework do not always align with manuscript styles. Another possible solution could be to situate manuscript writing as close as possible to the end of the term (prior to the next term). For the mid-year break, this may not be achievable given that academics are likely to be occupied with upcoming semester activities. This leaves only the longer summer break—a period of time when I, myself, might trial running some longer writing retreats, in the hope of greater publication success.

## Summary

There are many reasons to encourage undergraduates to publish: it expands their learning and employability; offers a chance to share findings; and (potentially) provides the supervising academic (if co-authoring) increased outputs. Yet, most students lack the awareness, motivation or skills necessary to publish from their research. To address this, I developed a model of *student writing weekends*, to guide students toward publication. The writing weekends offer students a small window of protected time to write in a supportive, encouraging, and dedicated environment, which serves to demystify the process of writing, and create some writing momentum. The writing weekends, in bringing like-minded students together, allow supervisors to more easily role model the value and responsibility of dissemination, and to co-write with their students. Nonetheless, as students move onto subsequent study, trying to publish from previous research project is not without its challenges. Even with those added complications, I would still encourage academic staff with multiple research students to try this model—adapted to their individual needs—for developing and jump-starting undergraduate publication capacity.

## Author Contributions

The author confirms being the sole contributor of this work and has approved it for publication.

### Conflict of Interest Statement

The author declares that the research was conducted in the absence of any commercial or financial relationships that could be construed as a potential conflict of interest.
